# Dysregulated expression of lipid storage and membrane dynamics factors in *Tia1* knockout mouse nervous tissue

**DOI:** 10.1007/s10048-014-0397-x

**Published:** 2014-03-23

**Authors:** Melanie Vanessa Heck, Mekhman Azizov, Tanja Stehning, Michael Walter, Nancy Kedersha, Georg Auburger

**Affiliations:** 1 Experimental Neurology, Department of Neurology, Goethe University Medical School, Building 89, 3rd floor, Theodor Stern Kai 7, 60590 Frankfurt am Main, Germany; 2 Institute for Medical Genetics, Eberhard-Karls-University of Tuebingen, 72076 Tübingen, Germany; 3 Division of Rheumatology, Immunology and Allergy, Brigham and Women’s Hospital, Smith 652, One Jimmy Fund Way, Boston, MA 02115 USA

**Keywords:** TIA-1, Transcriptome, Cell cycle, Lipid trafficking, RNA processing machinery, Motor neuron disease, Frontotemporal dementia, Cerebellar ataxia

## Abstract

During cell stress, the transcription and translation of immediate early genes are prioritized, while most other messenger RNAs (mRNAs) are stored away in stress granules or degraded in processing bodies (P-bodies). TIA-1 is an mRNA-binding protein that needs to translocate from the nucleus to seed the formation of stress granules in the cytoplasm. Because other stress granule components such as TDP-43, FUS, ATXN2, SMN, MAPT, HNRNPA2B1, and HNRNPA1 are crucial for the motor neuron diseases amyotrophic lateral sclerosis (ALS)/spinal muscular atrophy (SMA) and for the frontotemporal dementia (FTD), here we studied mouse nervous tissue to identify mRNAs with selective dependence on *Tia1* deletion. Transcriptome profiling with oligonucleotide microarrays in comparison of spinal cord and cerebellum, together with independent validation in quantitative reverse transcriptase PCR and immunoblots demonstrated several strong and consistent dysregulations. In agreement with previously reported *TIA1* knock down effects, cell cycle and apoptosis regulators were affected markedly with expression changes up to +2-fold, exhibiting increased levels for *Cdkn1a*, *Ccnf*, and *Tprkb* vs. decreased levels for *Bid* and *Inca1* transcripts. Novel and surprisingly strong expression alterations were detected for fat storage and membrane trafficking factors, with prominent +3-fold upregulations of *Plin4*, *Wdfy1*, *Tbc1d24*, and *Pnpla2* vs. a −2.4-fold downregulation of *Cntn4* transcript, encoding an axonal membrane adhesion factor with established haploinsufficiency. In comparison, subtle effects on the RNA processing machinery included up to 1.2-fold upregulations of *Dcp1b* and *Tial1*. The effect on lipid dynamics factors is noteworthy, since also the gene deletion of *Tardbp* (encoding TDP-43) and *Atxn2* led to fat metabolism phenotypes in mouse. In conclusion, genetic ablation of the stress granule nucleator TIA-1 has a novel major effect on mRNAs encoding lipid homeostasis factors in the brain, similar to the fasting effect.

**Electronic supplementary material:**

The online version of this article (doi:10.1007/s10048-014-0397-x) contains supplementary material, which is available to authorized users.

## Introduction

Cells have evolved various mechanisms to compensate different types of environmental stress like UV irradiation, oxidative stress, or heat. Cytoplasmic stress responses include the formation of stress granules (SGs) and processing bodies (P-bodies) [1–4]. During stress, most messenger RNAs (mRNAs) are removed from ribosomal translation, thus conserving energy and allowing stress-induced damage repair or degradation [5]. While SGs are thought to be a place where the bulk of mRNAs, as well as some proteins, undergoes storage and triage, P-bodies contain mRNAs dedicated for decay [6]. This is compatible with the observation that SGs contain mRNAs within stalled translation initiation complexes including 40S ribosomal subunits but are devoid of eIF2, whereas P-bodies contain multiple mRNA decapping enzymes [6]. Both SGs and P-bodies are dynamic structures that assemble and disassemble rapidly [7]. They share a common pool of components and can fuse to exchange mRNAs [2, 6, 8]. In contrast to P-bodies, SGs only exist transiently during stress conditions [6].

This formation of cytoplasmic SGs depends on the shuttling of the 43 kDa protein TIA-1 from the nucleus and on the aggregation of a C-terminal proteolytic TIA-1 fragment of 15 kDa that includes a glutamine-rich prion-related domain (PRD) [1, 9–11]. TIA-1 was initially identified as T-cell-restricted intracellular antigen 1 and was subsequently investigated particularly in immunological cell types [12]. It contains also three RNA-recognition motifs (RRM) and binds to adenine/uridine-rich elements (AREs) in the 3’-untranslated region of mRNAs. TIA-1 (gene symbol *TIA1*) and its homolog TIAR (gene symbol *TIAL1*) have roles not only in the nucleus for gene transcription and pre-mRNA splicing [13, 14], but also in the cytoplasm for mRNA stability and translation regulation [5, 15, 16]. TIA-1 is associated with diverse cell processes including inflammation [16], apoptosis [17], autophagy [18], and cell proliferation [18, 19].

The role of SGs in human pathology have become increasingly clear, since mutations in several SG components are responsible for hereditary degeneration syndromes of peripheral and central motor neurons, namely amyotrophic lateral sclerosis (ALS), spinal muscular atrophy (SMA), and frontotemporal dementia (FTD). SG component proteins with a causal role for motor neuron diseases include TDP-43 (gene symbol *TARDBP*) [20–23], FUS [24–26], ATXN2 [27–29], SMN [30, 31], Tau (gene symbol *MAPT*) [32, 33], HNRNPA2B1, and HNRNPA1 [34]. In the SG component ATXN2, the presence of a polyglutamine domain mutation may lead to pathogenic unstable expansions. Intermediate size ATXN2 expansions comprise a risk factor for ALS through mRNA-mediated TDP-43 interaction [27, 35–37], while larger polyglutamine expansions in ATXN2 lead to Levodopa-responsive Parkinsonism [38] or to prominent cerebellar involvement with later progression to a multisystem atrophy of the nervous system, known as spinocerebellar ataxia type 2 (SCA2) [39]. Like TIA-1, several of these RNA-binding proteins shuttle from the nucleus to the cytoplasm during cell stress, and for TDP-43, it is known that its cytoplasmic accumulation depends on cyclin-dependent kinases [40]. While the protein composition of SGs is under intense investigation [41], much work remains to be done for the identification of mRNAs regulated by SGs, particularly in the vulnerable nervous tissue.

While all of these disease-associated proteins and their target RNAs shuttle to preformed SGs, the initial stress-induced nucleation of SGs appears dependent on TIA1, TIAL1, TTP, G3BP1/2, and FMRP [10, 32]. G3BP1 deletion results in massive neuronal death during embryogenesis, suggesting that it has a developmental role independent from its role(s) in the stress response [42]. TIA-1 is well characterized as a SG-nucleating protein, and *Tia1* knockout (KO) mice not only exhibit grossly normal brain development, but also exhibit high embryonic lethality, consistent with dysregulation of a stress response [16]. We now used these mice for a transcriptome screen of nervous tissue at adult age, aiming to define the consequences of defective SG formation on RNA processing. The results confirm previous results obtained from human *TIA1* knock down experiments in HeLa cells about cell cycle regulator modulation [19]. Importantly, our data documented novel strong effects on lipid storage and membrane dynamics factors. These insights may help to understand the disordered mRNA regulation, which makes a major contribution to the pathology underlying motor neuron diseases [43, 44].

## Material and methods

### Animals

*Tia1* KO mice (bred into C57BL6/J background for more than 10 generations) were obtained from Harvard University, Dana Farber Cancer Institute. In these mice, homologous recombination of exon 4 results in a shortened *Tia1* mRNA and absence of the 43 kDa TIA-1 protein [16]. C57BL6/J wild-type (WT) mice from The Jackson Laboratory were used as control. The animals were housed and kept in individually ventilated cages under routine health monitoring until the appropriate adult ages at the FELASA-certified mfd Diagnostics GmbH in Wendelsheim, Germany. They were fed ad libitum, were bred in homozygous matings, and were sacrificed by cervical dislocation. Nervous tissues and liver were removed in minimal time, frozen in liquid nitrogen, and stored at −80 °C. Genotypes were controlled by tail biopsy and DNA analysis. DNA was isolated from tail biopsies of *Tia1* KO mice by Proteinase K (Ambion) treatment. PCR was performed using 50 ng DNA, 17 μl Pink Juice [125 μM Cresol Red sodium salt (Sigma Aldrich), 12.5 % 10× PCR buffer with 15 mM MgCl_2_ (Applied Biosystems), 250 μM dNTPs (Thermo Scientific), 25 % sucrose], 0.25 μl Taq Polymerase (AmpliTaq® DNA Polymerase, Applied Biosystems) and 1 μl of the primers KO1 *5′-GTCGTGACAAGCCACACTTG-3′* and KO2 *5′-AATTCCATCAGAAGCTTATCGAT-3′*. The following conditions were applied: initial denaturation at 94 °C for 2 min, 33 cycles of 94 °C for 15 s denaturation, 58 °C for 30 s annealing, 72 °C for 1 min elongation, and a final elongation step at 72 °C for 10 min. The predicted length of the KO allele is 400 bp. Genotypes were further confirmed by quantitative real-time reverse transcriptase polymerase chain reaction (qPCR) measurement of *Tia1* mRNA in the tissues under study. All procedures were in accordance with the German Animal Welfare Act, the Council Directive of 24 November 1986 (86/609/EWG) with Annex II and the ETS123 (European Convention for the Protection of Vertebrate Animals).

### Transcriptome profiling

The dissected tissues cerebellum, spinal cord, midbrain, and liver from *Tia1* KO mice and WT C57BL6/J mice at the age of 12 and 24 weeks (*n* = 3 vs. 3 mice/age) were sent to MFT Services (Tübingen, Germany). After RNA extraction, linear amplification and biotinylation of 100 ng of total RNA was performed with the GeneChip HT 3′IVT Express Kit (Affymetrix, Santa Clara, CA, USA) according to the manufacturer’s instructions. GeneChip HT Mouse Genome 430 2.0 Array Plates (Affymetrix) were used to hybridize fifteen micrograms of labeled and fragmented cRNA, to wash, stain, and scan automatically in a GeneTitan instrument (Affymetrix). Each of these oligonucleotide microarray chips is able to detect more than 39,000 transcripts with multiple probes for each mRNA. Visual inspection of scanned images was used to control for hybridization artifacts and proper grid alignment. AGCC 3.0 (Affymetrix) processed results were stored in CEL files. Further data analysis steps were carried out with the software platform R 2.14.0 and Bioconductor 2.14.0 [45]. First, the complete expression information from every chip was background corrected, quantile normalized, and summarized with Robust Multichip Average [46]. Empirical Bayes shrinkage of the standard errors was employed to derive the moderated *F*-statistic [47]. The resulting *p* values underwent multiple testing corrections according to “Benjamini-Hochberg” [48]. A decision matrix was produced through the function “decide tests” within the limma package, to attribute significant changes to individual contrasts. Thus, significant up- or downregulations were encoded by values of 1 or −1, respectively, to compare the consistency of significant expression changes across tissues and ages. All original transcriptome data were deposited at the public database Gene Expression Omnibus (GEO series accession # GSE54418, http://www.ncbi.nlm.nih.gov/geo/query/acc.cgi?acc=GSE54418).

### RNA isolation and expression analysis

RNA for qPCR expression analysis was extracted from cerebellar tissue (25 mg) of 12-week-old mice with Trizol® reagent (Invitrogen). Before cDNA synthesis, 1 μg of RNA was digested with DNase I Amplification Grade (Invitrogen). Reverse transcription was performed with SuperScript III Reverse Transcriptase (Invitrogen). Subsequently, expression levels were measured with the StepOnePlus Real-Time PCR System (Applied Biosystems) using 25 ng cDNA, 10 μl of FastStart Universal Probe Master (Rox) Mix (04914058001, Roche), and 1 μl of one of the following TaqMan Assays (Applied Biosystems): *Atxn2* (Mm01199894_m1), *Bid* (Mm00432073_m1), *Ccnf* (Mm00432385_m1), *Cdkn1a* (Mm00432448_m1), *Cntn4* (Mm00476065_m1), *Dcp1b* (Mm01183995_m1), *Inca1* (Mm01243670_m1), *Pabpc1* (Mm00849569_s1), *Plin4* (Mm01272159_m1), *Pnpla2* (Mm00503046_g1), *Tbc1d24* (Mm00557451_m1), *Tardbp* (Mm00523870_g1), *Tia1 Exon 3–4* (Mm01183616_m1), *Tial1* (Mm00437049_m1), *Tprkb* (Mm00616325_m1), *Tsen2* (Mm01184390_m1), *Wdfy1* (Mm00840455_m1), and *Tbp* (Mm00446973_m1) as endogenous control. The PCR conditions were 50 °C for 2 min, 95 °C for 10 min, and 40 cycles of 95 °C for 15 s and 60 °C for 60 s. Analysis of the gene expression data was conducted using the 2^−ΔΔCt^ method [49].

### Protein extraction and quantitative immunoblots

For SDS-PAGE followed by immunoblotting, protein was extracted from 25 mg cerebellar tissue of 12-week-old mice. The tissue was homogenized with a motor pestle in 10 vol. RIPA buffer [50 mM Tris–HCl (pH 8.0), 150 mM NaCl, 1 mM EDTA, 1 mM EGTA, 1 % Igepal CA-630 (Sigma), 0.5 % sodium deoxycholate, 0.1 % SDS, 1 mM PMSF, Complete Protease Inhibitor Cocktail (Roche)] and incubated on ice for 15 min. After centrifugation at 4 °C and 16,000×*g* for 20 min, the supernatant was stored (RIPA-soluble fraction), and the remaining pellet was dissolved in ½ vol. 2 × SDS buffer [137 mM Tris–HCl (pH 6.8), 4 % SDS, 20 % glycerol, Complete Protease Inhibitor Cocktail (Roche)] by sonification followed by 10 min of centrifugation at 16,000×*g*. The resulting supernatant was stored as RIPA-insoluble fraction. Protein concentration was determined with the BCA protein assay kit (Interchim, France), and 20 μg of each sample were loaded onto a 7.5 % polyacrylamide gel. After gel electrophoresis, the proteins were transferred to a PVDF membrane by wet blotting. The membranes were blocked with 5 % slim milk powder in PBST and incubated with antibodies against PLIN4 (1:500, Novus Biologicals), WDFY1 (1:500, Life Span BioSciences), CNTN4 (1:1,000, Abcam), or β-ACTIN (1:10,000, Sigma). ECL (Pierce) was used for visualizing the bands, which were subsequently quantified via densitometric analysis with ImageJ.

### Statistical analysis

Data were analyzed with GraphPad Prism software version 5.04 (2010) using Student’s *t* test. Error bars indicate SEM. Significant *p* values (<0.05) were marked as follows: *p* < 0.05 *, *p* < 0.01 **, *p* < 0.001 ***.

## Results

### Transcriptome survey identifies strong changes of specific mRNAs in spinal cord

Microarray chip profiling of the transcriptome detected the loss of *Tia1* correctly by one oligonucleotide (1431708_PM_a_at) corresponding to sequences at exon 4, whereas *Tia1* oligonucleotides covering exons 9–11 (1416813_PM_at, 1416812_PM_at, 1416814_PM_at, 1437934_PM_at) detected significant upregulation of expression. These observations are in good agreement with a previous report [16] stating that the homologous recombination event within the *Tia1* gene deletes sequences at exon 4, resulting in a shortened stable mRNA and in absence of TIA-1 protein. In the spinal cord, the expression profiling documented 115 oligonucleotides with significant upregulation both at 12 and 24 weeks of age vs. 70 oligonucleotides with significant downregulation at both ages, upon comparison of 3 KO and 3 WT tissues. The strongest three upregulations in spinal cord concerned *Plin4* (3.3-fold), *Wdfy1* (average 2.3-fold, detected consistently by three oligonucleotide spots), and *Cdkn1a* (average 2.2-fold, detected consistently by two oligonucleotide spots), while the strongest three downregulations concerned *Gkn3* (in human only a pseudogene is conserved [50]), *Bid* (−1.9-fold), and *Tsen2* (−1.8-fold) (Table 1). To further eliminate false positive candidates and to focus the investigation on mRNAs with relevance also for other tissues, the consistency of significant expression changes was compared from spinal cord to cerebellum, midbrain, and liver at both ages. Transcripts with significant expression change in the same direction in at least six out of the eight conditions under study were selected. They constituted 32 upregulations and 20 downregulations. All these *Tia1* KO transcriptome data were made publically available via the GEO database. We concentrated further research on 17 transcripts with known function in shared pathways (Table 1).

**Table 1:** Transcriptome profiling in four *Tia1* KO mice tissues at two ages identifies consistent expression dysregulations. *Tia1* KO and WT mice (3 vs. 3 at age 12 and 24 weeks) were compared, the significance of expression changes was determined, and consistently dysregulated transcript levels were shown with average fold changes. Negative values represent reduced expression (with *green color* highlighting its significance), while positive values represent induced expression (with *red color* highlighting its significance). *Bold values* illustrates transcripts with established induction by fasting conditions. The transcripts were grouped to reflect the convergent functions of the corresponding gene products in three pathways and were shown in alphabetical order

Gene symbol	Gene name	Oligo spot ID	Fold change			
			Spinal cord 12 weeks	Spinal cord 24 weeks	Cerebellum 12 weeks	Cerebellum 24 weeks
*Tia1*	Cytotoxic granule-associated RNA-binding protein 1 (TIA-1)	1431708_PM_a_at	*−4.78*	*−4.72*	*−3.60*	*−3.45*
Cell cycle control						
*Bid*	BH3 interacting domain death agonist	1417045_PM_at	*−1.98*	*−1.82*	*−1.67*	*−1.70*
*Ccnf*	Cyclin F	1422513_PM_at	*1.44*	*1.35*	*1.65*	*1.40*
*Cdkn1a*	Cyclin-dependent kinase inhibitor 1A (P21/Cip1)	1421679_PM_a_at	*1.83*	*2.48*	*3.07*	*1.98*
		1424638_PM_at	*1.88*	*2.57*	*2.74*	*1.88*
*Fgfrl1*	Fibroblast growth factor receptor-like 1	1447878_PM_s_at	*−1.36*	*−1.35*	*−1.21*	*−1.59*
*Inca1*	Inhibitor of CDK, cyclin A1 interacting protein 1	1448034_PM_at	*−1.28*	−1.15	*−1.26*	*−1.33*
*Nde1*	Nuclear distribution gene E homolog 1 (*A. nidulans*)	1435737_PM_a_at	*1.32*	*1.28*	*1.53*	*1.31*
*Tprkb*	Tp53rk binding protein	1425410_PM_at	*1.32*	*1.28*	*1.55*	*1.54*
Lipid storage and membrane trafficking						
*Angptl4*	**Angiopoietin-like 4**	1417130_PM_s_at	*2.30*	*2.50*	*2.05*	*1.79*
*Cntn4*	Contactin-4	1438782_PM_at	*−1.31*	*−1.50*	*−2.32*	*−2.61*
*Mfsd2a*	**Major facilitator superfamily domain containing 2A**	1428223_PM_at	*1.51*	*1.37*	*1.58*	*1.49*
*Plin4*	Perilipin-4	1418595_PM_at	*3.51*	*3.05*	*2.62*	*2.11*
*Pnpla2*	Patatin-like phospholipase domain containing 2	1428143_PM_a_at	*1.42*	*1.41*	*1.40*	*1.20*
*Pnpla7*	**Patatin-like phospholipase domain containing 7**	1451361_PM_a_at	*1.24*	*1.42*	*1.28*	*1.37*
*Tbc1d24*	TBC1 domain family, member 24	1448028_PM_at	*1.68*	*1.42*	*1.95*	*1.54*
		1442325_PM_at	*1.97*	*1.46*	*1.73*	*1.55*
*Wdfy1*	**WD repeat and FYVE domain-containing 1**	1424749_PM_at	*1.36*	*3.15*	*1.38*	*2.94*
		1437358_PM_at	*1.37*	*3.32*	*1.31*	*2.91*
		1435588_PM_at	*1.34*	*3.17*	*1.39*	*2.75*
RNA processing machinery						
*Dcp1b*	DCP1 decapping enzyme homolog b (*S. cerevisiae*)	1444030_PM_at	*1.93*	*1.94*	*2.73*	*1.71*
*Tsen2*	tRNA splicing endonuclease 2 homolog (*S. cerevisiae*)	1459346_PM_at	*−1.66*	*−1.87*	*−1.83*	*−1.67*

### qPCR validates dysregulated levels of several transcripts in three pathways

Convergent effects were evident for the pathways of lipid storage and membrane trafficking, of cell cycle control, and additionally of the RNA processing machinery. The changes in expression levels of such genes were reassessed by the independent technique qPCR in cerebellum (Suppl. Figure 1). The results on the lipid pathway confirmed upregulations for *Plin4* which encodes a lipid droplet storage factor (3.2-fold), for *Wdfy1* encoding a modulator of PI3K control over endosome membrane trafficking (3.2-fold), for *Tbc1d24* as Rab-GTPase activating vesicle dynamics factor (2.1-fold), and for *Pnpla2* as component of the lipolytic cascade and as regulator of adiposome size (1.5-fold). A membrane pathway relevant downregulation was observed for *Cntn4* as a glycosylphosphatidylinositol-anchored membrane adhesion factor implicated in axon network connections and synaptogenesis (−2.4-fold). Regarding the cell cycle pathway, upregulations were confirmed for *Cdkn1a* as cycle progression inhibitor (1.5-fold), *Ccnf* as a centrosome reduplication inhibitor during G2 phase (1.6-fold), and *Tprkb* as an ADP-ribose activated and p53-related protein kinase that transduces the PI3K/TOR pathway (1.1-fold). Cell cycle pathway relevant downregulations were confirmed for *Bid* as an ATM-effector that also activates the S-phase checkpoint (−1.7-fold), and *Inca1* as an interactor of cyclin A1 that inhibits cyclin-dependent kinase and proliferation (−1.3-fold). Regarding the RNA processing pathway, the upregulation was confirmed for *Dcp1b* (1.2-fold) as a component of the RNA decapping and degradation machinery in P-bodies. In contrast, for *Tsen2*, the qPCR results suggested a significant upregulation (1.1-fold) instead of the downregulation previously observed by oligonucleotide microarray chips, a puzzling result since alternative splicing isoforms for this transcript are not documented. Since microarray chip data depend on the oligonucleotide choice and quality, additional hypothesis-driven qPCR were performed for important SG components with relevance for neurodegeneration and general mRNA translation. These experiments revealed a significant increase in the levels of *Tial1* (1.2-fold), but did not detect major changes for the *Pabpc1*, *Tardbp*, or *Atxn2* transcript levels. Altogether, most strong candidates from the transcriptome screening could be validated upon individual reassessment.

### Quantitative immunoblots demonstrate altered levels for PLIN4, WDFY1, and CNTN4

**Fig. 1:**
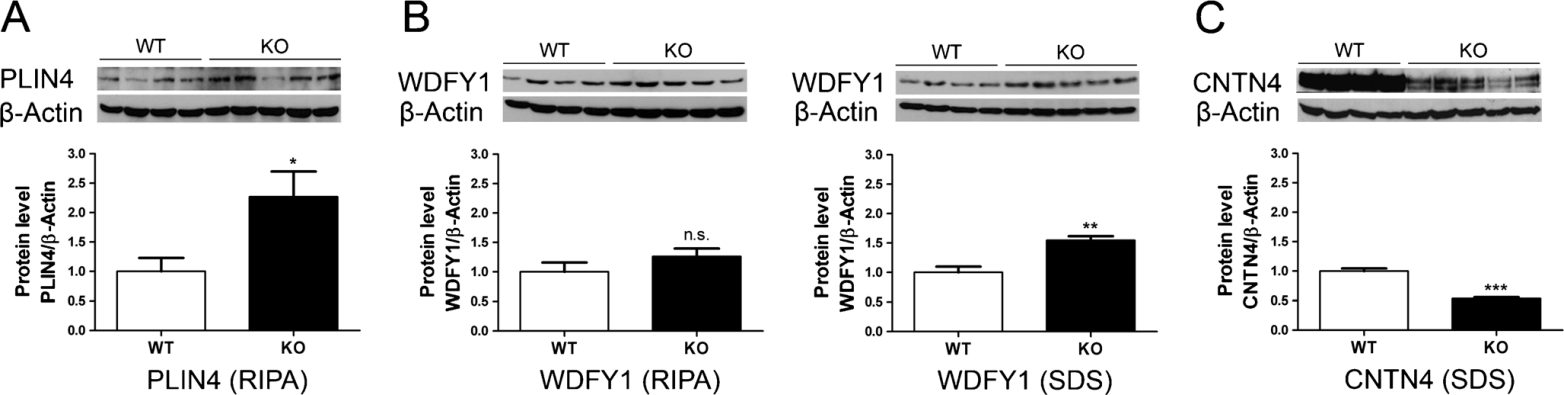
Quantitative immunoblots demonstrate significantly increased levels of perilipin-4 and WDFY1, but decreased levels of CNTN4 in *Tia1* KO tissue. In cerebellum of 12-week-old mice (**a**), the PLIN4 levels were elevated in the RIPA-soluble protein fraction, whereas (**b**) the WDFY1 levels were elevated in the SDS fraction and (**c**) the CNTN4 levels were decreased in the SDS fraction (*n* = 4 WT vs. 5 KO mice)

To test whether these alterations of mRNA levels are compensated, for example by increased translation rates, or possess downstream consequences for the respective protein levels, quantitative immunoblots of cerebellar tissue were performed for three factors in the membrane dynamics pathway. Corresponding to the upregulation of the *Plin4* transcript, the perilipin-4 protein levels were significantly upregulated (2.2-fold) in the RIPA-soluble tissue fraction that contains the freely soluble proteins (Fig. 1a), while they were undetectable in the SDS-soluble tissue fraction that contains membranes and more insoluble proteins. This observation is consistent with previous reports that perilipin-4 is recruited onto ER-membranes and lipid droplets only when factors such as diacylglycerol become abundant [51]. Again, in parallel to the upregulation of the *Wdfy1* transcript, the WD repeat and FYVE domain-containing 1 protein levels were significantly upregulated (1.5-fold) in the SDS-soluble tissue fraction, while its presence in the RIPA-soluble tissue fraction was not significantly altered (Fig. 1b). The localization of WDFY1 to the SDS fraction is consistent with the FYVE domain association with the phosphatidylinositol 3-phosphates of endosomal membranes [52]. In agreement with the downregulation of the *Cntn4* transcript, the contactin-4 protein levels were significantly decreased (−1.9-fold) in the SDS-soluble tissue fraction (Fig. 1c). Thus, the *Tia1* knockout has selective effects on mRNA levels, resulting in abnormal levels of at least three proteins in the pathway of membrane dynamics and lipid storage.

## Discussion

In the past, transcriptome profiling has been helpful to document changes in overall transcription and RNA processing, leading to the discovery of altered pathways and signaling networks in human disease [53]. While it is usually cumbersome in an organism to unravel how stressors impact neuronal function in molecular detail, this study of knockout tissues identifies novel selective RNA effects of TIA-1, which cause altered levels of the corresponding proteins that modulate membrane dynamics and lipid storage.

TIA-1 is a key stress granule component, capable of nucleating SGs when overexpressed and inhibiting SG formation when absent [10]. As a consequence, one might have expected an alteration in the levels of other stress granule components when TIA-1 is depleted. However, this assumption was not corroborated in the *Tia1* KO mouse tissues for most of the SG-associated genes tested. In the transcriptome data, there was no obvious dysregulation for any other known SG components. There are several possible explanations for this: (1) the loss of *Tia1* might be compensated by expression changes in other genes that were not present on the chip, by alternative splicing changes that are not represented on the microarray chip or by expression changes with bare significance (e.g. *Tial1*); (2) a *Tia1* KO could have severe effects on the localization of stress granule components without influencing their expression; or (3) *Tia1* deletion might only have an effect on their expression levels under acute stress, which was absent from the tissue of young mice that were kept in a pathogen-free environment and were allowed to eat ad libitum. The slight upregulation of *Tial1* mRNA levels is probably a compensatory effort, since TIA-1 overexpression was observed to substitute for *Tial1* deletion and to correlate inversely with *Tial1* expression levels [54]. Interestingly, a relatively stronger upregulation of *Dcp1b*, encoding a core component of the mRNA decapping complex in P-bodies, may indicate increased mRNA decay in the absence of TIA-1.

The more substantial effects of the *Tia1* KO on cell cycle and apoptosis-related factors are in agreement with previous reports [19]. A team investigating the effects of *TIA1* knock down in human HeLa cells observed proliferative effects with increased cell numbers in S- or G2/M-phases and an induction of anchorage-independent growth, in parallel to upregulation of interleukin/chemokine transcripts and downregulation of transcript levels for the tumor necrosis factor superfamily member 10 and the P21protein/CDKN1A-activated kinase PAK3 [19]. In partial accord, a recent study of *Tia1* KO effects in murine embryonic fibroblasts observed again a prominent cell cycle effect, but documented reduced rates of cell proliferation, cell cycle progression delay, increased cell size, and apoptosis enhancement [18]. Our data documented downregulated transcript levels for apoptosis-promoting factors such as *Bid* and *Fgfrl1*. The downregulation of *Bid* was previously described to occur after serum starvation and to induce autophagy [55, 56]. The downregulated transcript levels of cell cycle inhibitors such as *Fgfrl1* and *Inca1* on the one hand, together with the upregulated transcript levels of cell cycle enhancers like *Ccnf* and *Nde1* transcripts, seem difficult to integrate with the upregulation of the cell cycle inhibitor *Cdkn1a* on the other hand. Beyond possible consequences for neurogenesis, there is a clear role of CDKN1A/p21 for glia proliferation [57]. The upregulation of CDKN1A expression is a known response to starvation, which arrests the cell cycle and thus protects from cell death [58, 59]. Beyond glia cells, an additional role of CDKN1A/p21 in adult neurons regarding DNA damage response, neuroprotection, neuronal senescence, motor neuron regeneration, and tauopathy is established [60–66]. In this context, also the upregulation of *Nde1* is interesting, since it encodes a modulator of mitotic spindle function and progenitor migration, which is responsible for neuron number in cortical layers II-IV [67]. Altogether, the role of TIA-1 for regulating cell cycle, cell death, and stress responses in adult nervous tissue is credible.

Our transcriptome profiling highlighted an unknown function for TIA-1 in membrane dynamics and lipid storage. One-fifth of the altered transcripts detected are involved in lipid storage, transport, or membrane trafficking, a number far exceeding stochastic expectations even in view of the high lipid content of brain tissue. Several dysregulated factors are involved in the formation of lipid droplets. These structures store neutral lipids in their core and are important for lipid transportation [68], vesicle trafficking, and cell signaling [69]. Perilipin 4 (encoded by *Plin4*) was shown in adipocytes to coat the nascent lipid droplets [70]. Accordingly, an upregulation of *Plin4* in the *Tia-1* KO mice might correlate with an enhanced formation or turnover of lipid droplets. This notion is strengthened by the fact that two other lipid droplet components, *Pnpla2* and *Pnpla7* (encoding patatin-like phospholipase domain containing 2 and 7, respectively) also show increased transcript levels. *Pnpla2* hydrolyzes triglycerides, thus providing the organism with energy through the supply of free fatty acids and altering membrane composition [68, 71]. This mechanism becomes important during starvation stress. Furthermore, it has been shown that *Pnpla7* levels are increased by fasting and that PNPLA7 may be involved in organophosphorus compound-induced motor neuron degeneration [72, 73]. Although our animals were not fasting, two other transcripts that are normally increased under this condition were also upregulated, namely *Mfsd2a* and *Angptl4* [74–76]. These data suggest that there are fasting-like stress conditions in the *Tia1* KO mouse model, which are independent of food availability, but balance the organism towards gaining energy from fatty acids. Thus, deletion of *Tia1* increases the levels of transcripts that are normally induced by fasting conditions and are involved in lipid transport and membrane trafficking.

The *Tia1* KO-induced upregulation of *Wdfy1* and downregulation of *Cntn4* levels modulate two factors implicated in phosphoinositide-dependent membrane binding. The WD repeat and FYVE domain-containing 1 protein interacts with phosphoinositide-3-phosphate enriched endosomal membranes, in particular under stress-induced acidic conditions, helping to recruit other proteins involved in membrane trafficking [52, 77]. Upregulation of *Wdfy1* can be induced by pharmacological inhibition of autophagy during starvation stress [78]. Interestingly, *Wdfy1* level upregulation and *Tia1* dysregulation were among the 16 most promising biomarkers that characterized the brain of mouse model of Alzheimer’s disease, with *Wdfy1* showing the changes earlier than *Tia1* [79]. Similarly, the upregulation detected consistently by two oligonucleotide spots for *Tbc1d24* encodes an activator of small GTPases involved in the regulation of membrane trafficking, which was shown to act as potent modulator of primary axonal arborization [80, 81]. Its homolog *Tbc1d1* was linked to human obesity and a *Tbc1d1* mutation underlies the absence of diet-induced obesity in the lean mouse strain [82–84]. A perhaps even more intriguing finding regarding medical relevance is the downregulation of contactin-4, since this glycosylphosphatidylinositol-anchored neuronal adhesion protein is involved in axon guidance and synaptic plasticity [85–88] and interacts with the Alzheimer’s disease mediator amyloid precursor protein [89]. Genetic haploinsufficiency of contactin-4 was demonstrated to cause developmental delay [90]. Other members of the contactin protein family have been implicated in selective motor neuron pathology, namely contactin-1 in human [91] and the contactin-2 ortholog in zebrafish [92, 93]. It is noteworthy that contactin-2/TAG1 is a strong regulator of diet-induced obesity [94]. Thus, these data emphasize the role of TIA-1 for the stress-dependent composition and trafficking of membranes as well as their protein interactions.

It is important to note that the effect of TIA-1 on lipid and membrane dynamics is paralleled by similar effect of two other SG components. A genetic ablation of the RNA-binding protein ATXN2 in mice leads to obesity, appearance of lipid droplets in the liver, increased blood cholesterol, cerebellar gangliosides, and sulfatides [95]. Conversely, gain-of-function mutations of ATXN2 lead to a multisystem atrophy of the nervous system [39]. This scenario with ATXN2 loss-of-function affecting lipid homeostasis, while its excess causes neurodegenerative diseases, shows a striking similarity to the effects of TDP-43. Postnatal deletion of the TDP-43-encoding *Tardbp* gene was shown to cause dramatic loss of body fat and weight together with a downregulation of the leanness factor *Tbc1d1* [96]. Conversely again, the overexpression of *Tardbp* leads to increased fat deposition and adipocyte hypertrophy together with an upregulation of *Tbc1d1* [97]. A representative TDP-43 mutation that causes neurodegenerative diseases was shown to enhance normal TDP-43 splicing function for some RNA targets but loss-of-function for others, in the absence of aggregation or nuclear depletion of TDP-43 [98]. Jointly, these data underscore a prominent role of three SG components for mRNAs that regulate lipid metabolism and membrane composition under stress.

In conclusion, our data show that ablation of *Tia1* in mouse tissues leads to changed expression levels of few constituents of the mRNA processing machinery, of specific cell cycle and apoptosis pathways components, and of various lipid storage and membrane dynamics factors. We propose that TIA-1 depletion induces starvation-like conditions as a trigger for the upregulation of these transcripts. These findings may be relevant to elucidate the role of stress granules and aberrant RNA processing for the prominent axon transport pathology in motor neuron diseases such as ALS, SMA, FTD, and SCA2.

## Electronic supplementary material

Below is the link to the electronic supplementary material.

Supplementary Figure 1 qPCR analysis of cerebellar expression in 12-week-old WT and *Tia1* KO mice validates most microarray findings. Several transcripts involved in (A) cell cycle regulation, (B) lipid storage and membrane dynamics, and (C) RNA processing were demonstrated to show significant expression changes (n = 6 WT vs. 6 KO mice, the order within each pathway is alphabetical). The >3-fold upregulation of *Plin4* and *Wdfy1* and >−2-fold downregulation of *Cntn4* transcript levels were prominent. (JPEG 1581 kb)

## Abbreviations

*Angptl4*: Angiopoietin-like 4
*Atxn2*: Ataxin-2
*Bid*: BH3 interacting-domain death agonist
*Ccnf*: Cyclin F
*Cdkn1a*: Cyclin-dependent kinase inhibitor 1A (P21/Cip1)
*Cntn4*: Contactin-4
*Dcp1b*: DCP1 decapping enzyme homolog B (*S. cerevisiae*)
*Fgfrl1*: Fibroblast growth factor receptor-like 1
*Inca1*: Inhibitor of CDK cyclin A1 interacting protein 1
*Mfsd2a*: Major facilitator superfamily domain containing 2A and angiopoietin-like 4
*Nde1*: Nuclear distribution gene E homolog 1 (NudE neurodevelopment protein 1)
*Pabpc1*: Poly(A)-binding protein cytoplasmic 1
*Plin4*: Perilipin-4
*Pnpla2*: Patatin-like phospholipase domain containing 2
*Pnpla7*: Patatin-like phospholipase domain containing 7
*Tbc1d24*: TBC1 domain family member 24
*Tbp*: TATA-box-binding protein
*Tardbp*: TAR DNA-binding protein-43 (TDP-43)
*Tia1*: T-cell-restricted intracellular antigen-1 cytotoxic granule-associated RNA-binding protein (TIA-1)
*Tial1*: TIA1 cytotoxic granule-associated RNA binding protein-like 1
*Tprkb*: Tp53rk binding protein
*Tsen2*: tRNA splicing endonuclease 2 homolog (*S. cerevisiae*)
*Wdfy1*: WD repeat and FYVE domain-containing 1 (FENS-1)
