# Supplementary figures and images for: Dysregulated expression of lipid storage and membrane dynamics factors in Tia1 knockout mouse nervous tissue
Source: Neurogenetics. 2014 Mar 23;15(2):135–44. doi: 10.1007/s10048-014-0397-x (PMC3994287; doi:10.1007/s10048-014-0397-x)

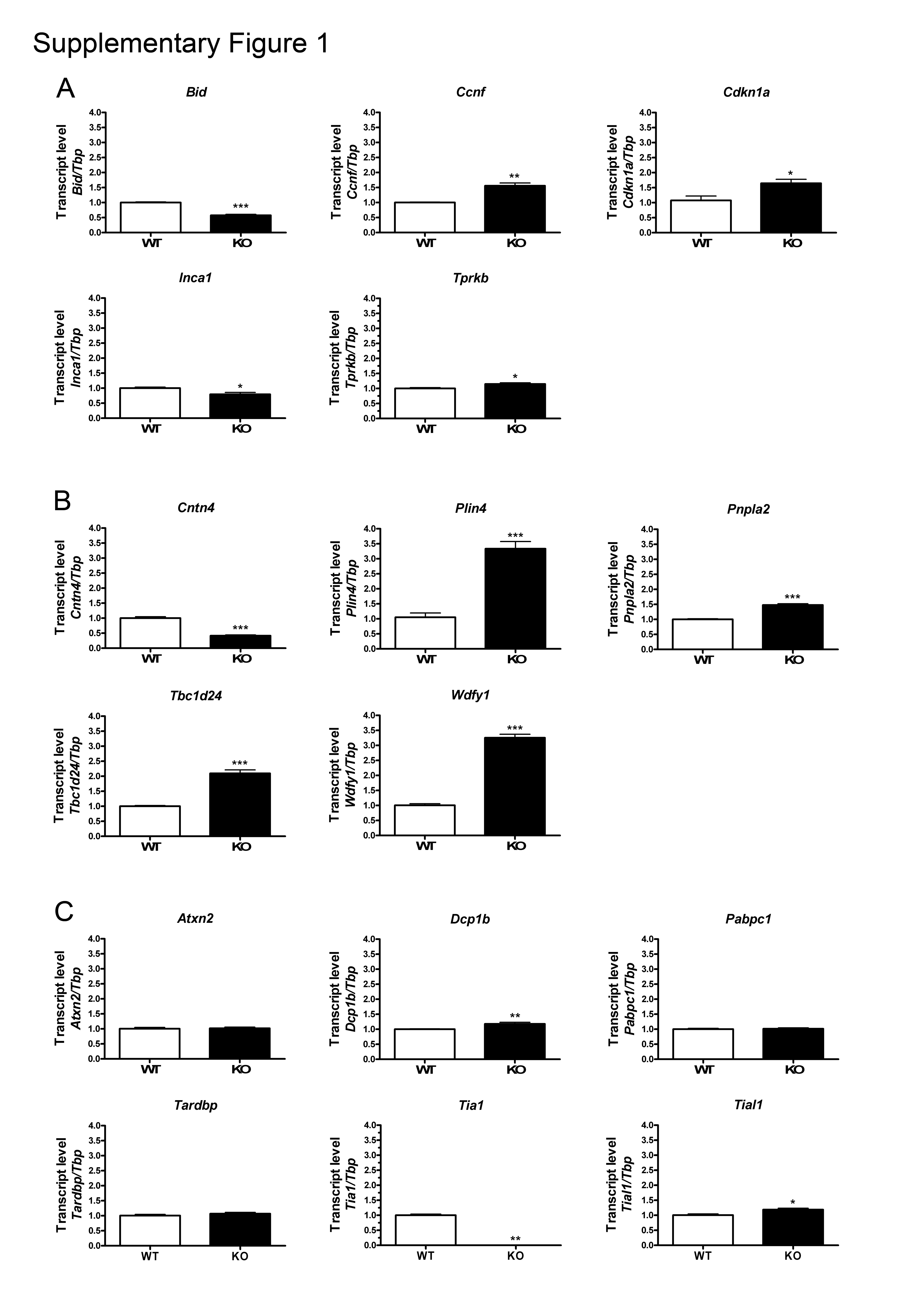

Supplement: Supplementary file 1 — qPCR analysis of cerebellar expression in 12-week-old WT and Tia1 KO mice validates most microarray findings. Several transcripts involved in (A) cell cycle regulation, (B) lipid storage and membrane dynamics, and (C) RNA processing were demonstrated to show significant expression changes (n = 6 WT vs. 6 KO mice, the order within each pathway is alphabetical). The >3-fold upregulation of Plin4 and Wdfy1 and >−2-fold downregulation of Cntn4 transcript levels were prominent. (JPEG 1581 kb) [file 10048_2014_397_Fig2_ESM.jpg]
